# Experiences of End-of-Life Decision-Making in Equine Veterinary and Charity Teams

**DOI:** 10.3390/ani15050678

**Published:** 2025-02-26

**Authors:** Amelia Cameron, Megan Geldard, Tim Mair, Gary England, John Burford, Sarah Freeman, Kristian Pollock, Eleanor Wilson

**Affiliations:** 1 School of Veterinary Medicine and Science, University of Nottingham, Loughborough LE12 5RD, UK; amelia.cameron1@nottingham.ac.uk (A.C.); svymg3@nottingham.ac.uk (M.G.); john.burford@nottingham.ac.uk (J.B.); sarah.freeman@nottingham.ac.uk (S.F.); 2 Bell Equine Veterinary Clinic, CVS Group Ltd., Mereworth ME18 5GS, UK; tim.mair@btinternet.com; 3 Nottingham Centre for the Advancement of Research into Supportive, Palliative and End of Life Care, School of Health Sciences, University of Nottingham, Nottingham NG7 2HA, UK; kristian.pollock@nottingham.ac.uk (K.P.); eleanor.wilson@nottingham.ac.uk (E.W.)

**Keywords:** euthanasia, mental health, communication, shared decision-making, equine welfare, vet-client interactions

## Abstract

Veterinary and charity teams are frequently involved in equine end-of-life decisions. These can cause ethical dilemmas and emotional burdens and complicate communication with owners. Semi-structured focus groups explored experiences of making decisions about end-of-life care and euthanasia at three UK equine veterinary practices and one charity. There were 26 participants in a mix of roles. Inductive coding was conducted during thematic analysis. Major themes generated were ‘Equine Welfare’, ‘Relationships’, ‘Decision-Making’, and ‘Emotional Toll and Coping Strategies’. Communication, finances, and chronic vs. acute cases were key issues featured within multiple themes. The individual horse’s welfare was the participants’ priority during decision-making. However, there were barriers to coming to a decision about treatment or euthanasia, and conflict caused by these barriers could detrimentally affect participants’ mental health. Preferred style of decision-making varied depending on the situation. Examples given included shared decision-making, participants steering the decision, and attempts by participants or owners to shift decisional responsibility onto the other party. Some owners sought additional input into decisions from veterinary nurses and receptionists. Participants found aspects of their roles emotionally challenging. This could be mitigated by supportive relationships and communication with colleagues. Involving veterinary team members in varying roles in end-of-life care planning and euthanasia decision-making with owners contributes a range of skills and expertise and helps to share the burden of responsibility.

## Simple Summary

People working in equine veterinary practices and charities often contribute to euthanasia decisions for horses. Making these decisions can be stressful and affect mental health. Focus groups were conducted with different professionals working in a mix of roles at three UK equine veterinary practices and one charity. Participants were asked about their experiences of contributing to euthanasia decisions. They reported that they prioritised the welfare of the horses under their care but that they encountered barriers to making treatment or euthanasia decisions that impacted welfare. This could negatively affect mental health. Participants favoured different styles of decision-making depending on the situation, such as joint decisions vs. one person taking the lead. Sometimes, veterinary nurses and receptionists also influence decision-making, as well as veterinary surgeons. Although participants found aspects of their roles emotionally challenging, having support from colleagues could help to ease the burden. Collaborative planning and decision-making for horses’ end-of-life between the owner, veterinary surgeon, and other members of the veterinary team, where appropriate, would allow the contribution of a range of knowledge and skills.

## 1. Introduction

Veterinary practitioners face regular euthanasia-related ethical dilemmas which cause stress, including convenience euthanasia, owner financial limitations, and owners wishing to continue treatment despite poor quality of life (QOL) [1]. Delayed euthanasia has been recognised as a prevalent equine welfare issue with the potential to cause great suffering [2]. This is where an animal has experienced prolonged and unnecessary suffering, which cannot be alleviated, as a result of not being euthanased sooner. Owners have the authority to make the final decision whether to euthanase their animal or not, while veterinary surgeons can offer owners their advice if they believe euthanasia should be considered [3]. In contrast, convenience euthanasia is where an animal is euthanased for the benefit of humans, and it is not deemed necessary from a welfare perspective [1]. The Royal College of Veterinary Surgeons defines euthanasia as “painless killing to relieve suffering” [3]. However, in the UK, animals are considered property, and so can be euthanased at the request of the owner for any reason [4]. However, veterinary surgeons can refuse if they do not believe euthanasia is necessary [3].

Horses can be regarded as companion animals or livestock depending on the context, though, in the UK, the majority of owners consider their horses to be pets and/or family members [5]. Therefore, research into the human–animal relationships of small companion animal and equine owners is regularly comparable. Companion animal owners frequently expect their veterinary surgeons to provide emotional support when making and enacting euthanasia decisions [6]. However, veterinary professionals often find giving this empathy and support emotionally difficult, which increases their risk of burnout and compassion fatigue [7,8]. Many veterinary professionals have also reported feeling underprepared to have end-of-life discussions with owners and unsure of how best to support them [7,8]. Support from veterinary surgeons can decrease owners’ negative emotions associated with grief after euthanasia [9]. Poor communication or a lack of empathy can negatively impact owners, for example, by increasing feelings of guilt or bereavement [10]. It is not just veterinary surgeons who are affected, veterinary nurses and receptionists also communicate with owners about end-of-life [11]. However, research into the roles of veterinary nurses and receptionists is lacking compared with research focusing on veterinary surgeons [12]. A survey involving veterinary team members in a range of roles found that high team effectiveness resulted in increased job satisfaction, while a negative team environment contributed to burnout [13]. This highlights the importance of research that explores the perspectives and experiences of a wide range of team members.

There is a large population of unwanted horses in the UK, and the majority of charities are already full or overstretched [14]. Therefore, equine charity teams will also commonly encounter euthanasia and likely face similar ethical challenges. Animal shelter workers have been identified as being at high risk of poor mental health and compassion fatigue, due to factors such as frequent exposure to animal suffering and euthanasia [15]. Some horses may remain at charities long term, meaning staff have the potential to develop strong relationships with them, which may make euthanasia decisions more difficult [5]. Multiple team members may also be involved in care and end-of-life decisions for equine residents. Additionally, charities may have horses that are rehomed or on loan, which they monitor. This means that charity veterinary practitioners or other team members may need to facilitate end-of-life discussions and make euthanasia decisions with loaners or rehoming owners. Due to the variety of people in different roles that may contribute to end-of-life decisions for rescued horses, it would be beneficial to explore the decision-making strategies employed and compare these to veterinary practice. Research exploring this is currently lacking, and there may be complementary and transferable approaches between these different settings.

Several strategies exist in human medical decision-making. Traditionally the paternalistic approach was taken, where the health care provider makes decisions and directs the patient/caregiver, with the assumption their expertise makes them best placed to take responsibility [16]. The autonomous approach was developed in response to changing attitudes that patients should be able to decide on the best option for themselves [16]. In this case, the health care provider explains the options and shares their medical knowledge to facilitate the patient making an informed decision but aims not to influence what option they choose [16]. Shared decision-making (SDM) was also developed to challenge the paternalistic model. The reason is that patients may not want to be responsible for making medical decisions on their own, but they do want their input into decisions to be valued [16,17]. SDM promotes active collaboration between patients and healthcare providers and has been widely promoted as the best model in human medicine [17]. It has also become increasingly recommended in veterinary medicine [18]. Limited research currently exists on decision-making approaches and preferences in equine veterinary and charity settings. Gaining an understanding of these will help to target new resources to support veterinary teams and owners. This study aimed to explore UK equine veterinary and charity teams’ experiences of making end-of-life decisions and the engagement and involvement of different team members. It also aimed to explore opinions on the use of resources to support decision-making and identify if any further resources may be beneficial.

## 2. Materials and Methods

The Consolidated Criteria for Reporting Qualitative research (COREQ) guidelines have been used as a reporting framework for this study [19].

### 2.1. Study Design

Four in-depth semi-structured focus groups were used to collect data that were analysed using thematic analysis [20]. A qualitative methodology was chosen as it allows in-depth data to be gathered on complex issues within a specific context, such as why people hold particular opinions or behave in a certain way [20,21].

### 2.2. Recruitment

A convenience sample of three UK equine veterinary practices and one UK equine charity was gathered by directly emailing representatives known to the authors and through advertising on Facebook. One practice and one charity contacted directly were unable to participate because of staff shortages due to requirements for contacts of COVID-19 cases to isolate. Participants in a mix of roles from each establishment were required. The representatives contacted were sent an invitation email, participant information sheet, and online consent form. They shared these with team members, who could sign up using the form. All participants were required to be at least 18 years old. Participation was voluntary, and no financial incentives were offered. It was decided to include an equine charity as a comparison of the settings in which euthanasia decisions can be made and to obtain an idea of the similarities and differences between how decisions might be made here compared to in the veterinary practices included.

### 2.3. Participants

There were 26 participants, with 5–8 in each focus group in a mix of roles and experience levels, from the same establishment. In the veterinary practice focus groups, participants included veterinary surgeons, veterinary nurses, and reception or office administration staff. Equine charity participants included a veterinary surgeon and a mix of staff in management roles, either directly involved in end-of-life decision-making or with other roles such as creating end-of-life policy or public-facing resources. Grooms were eligible to participate, but none signed up. Table 1 shows the roles of participants in each group. The veterinary practices were equine hospitals that offered ambulatory services. The charity was involved in euthanasia decisions for horses at the centre and with people who rehome charity horses and have them on loan. There are a small number of UK equine charities, so, for anonymity, specific charity staff roles have not been identified, other than that of a veterinary surgeon. All establishments have been given pseudonyms, with ‘Poppy Ridge Charity’ representing the equine charity. Horses referred to by participants have also been given pseudonyms. Two focus groups were held in-person at the practices, while the other two were held online (Whitedale, Poppy Ridge Charity) using Microsoft Teams (Version 1.4.00.22472) due to precautions against COVID-19. There was a distribution of male and female participants across the groups, though there was a higher proportion of female participants.

### 2.4. Data Collection

Interview schedules (Supplementary Materials File S1) were developed which included open questions and potential prompts. A pilot focus group was conducted, and feedback was used to amend the schedule.

Two vignettes involving euthanasia scenarios were developed and used in each group to stimulate discussion and elicit participants’ opinions and experiences [20], one of an emergency case and the other involving a chronic case (Supplementary Materials File S2). In-person focus groups were voice-recorded using portable voice recorders. Online focus groups were video- and audio-recorded through Microsoft Teams and with a portable voice recorder. All lasted 50–90 min and took place between August and December 2021.

All focus groups were conducted and analysed by the primary researcher (AC). A reflexivity statement is given in Supplementary Materials File S3.

### 2.5. Data Analysis

#### 2.5.1. Transcription

All veterinary practice focus groups were transcribed by AC. The charity focus group was transcribed by MG. Names and other potentially identifying content were removed. Transcripts were not reviewed by participants prior to or after coding.

#### 2.5.2. Coding and Theme Generation

Coding and data management were conducted on NVivo (Version 12, Lumivero, Denver, CO, USA). All transcripts were coded by AC; double coding was undertaken by KP and EW on all transcripts and MG on the charity transcript. Codes were discussed and refined between researchers. Codes were generated inductively, and all transcripts were coded iteratively by the primary researcher and then reviewed when a new code was generated and added to the codebook. The coding framework became stabilised during analysis, so no new codes were added.

Related codes were grouped to generate themes and subthemes. One sheet of paper (OSOP) analysis was completed for each group of codes, which involved mapping all the issues raised by the coded extracts in the group onto a single piece of paper to create a summary [22]. Each OSOP was developed into a subtheme by considering how the issues raised in the codes were related. These subthemes were then grouped and shaped into major themes. Selected quotes, presented verbatim other than minor edits to improve readability, have been used to illustrate points.

## 3. Results

### 3.1. Overview

**Figure 1:**
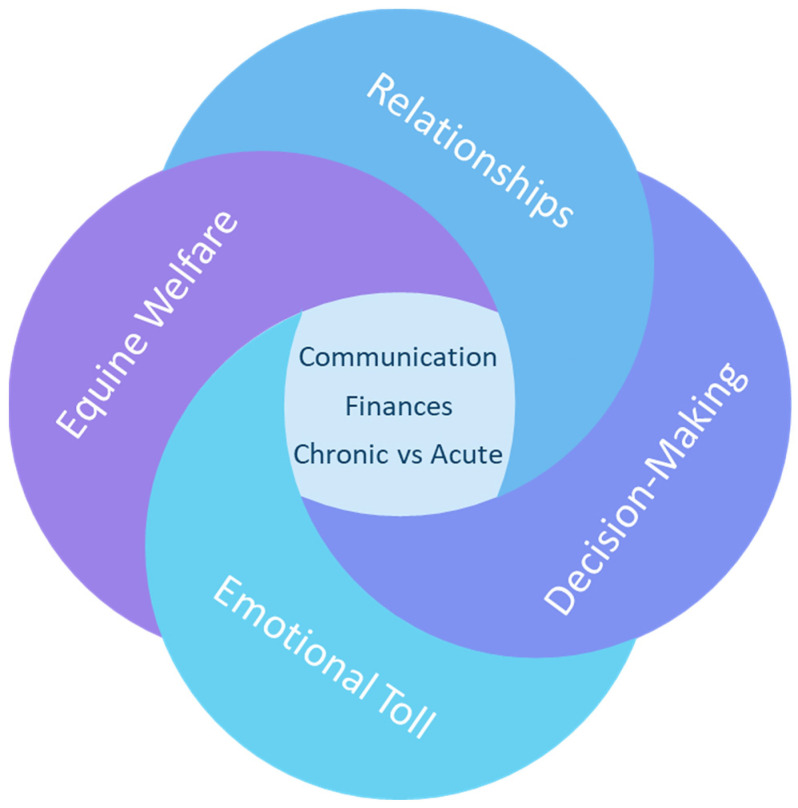
Diagram illustrating the interactions between the key themes generated during thematic analysis, and core issues featuring within these themes.

Thematic analysis generated four major interrelated and overlapping themes, ‘equine welfare’, ‘relationships’, ‘decision-making’, and ‘emotional toll and coping strategies’ (Figure 1). Each theme incorporates several subthemes. Core issues that featured across multiple themes were communication, finances, and differences between chronic and acute cases. These issues impacted how people made decisions, built relationships, approached equine welfare, and coped with difficult emotions.

### 3.2. Equine Welfare

**Figure 2:**
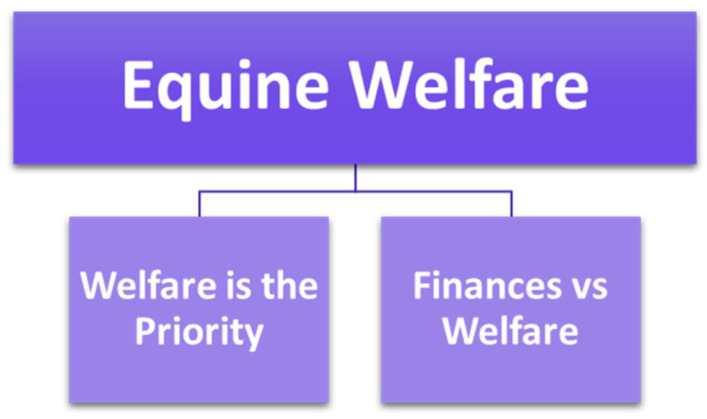
Diagram showing the theme ‘Equine Welfare’ and associated subthemes.

Figure 2 displays the subthemes within the theme “Equine Welfare”.

#### 3.2.1. Welfare Is the Priority

Participants frequently emphasised that the equine patient’s welfare was their priority when making decisions about treatment and euthanasia with owners. They described how it was important to avoid decisions being delayed too long which could lead to unnecessary suffering. Participants agreed that most owners they encountered prioritised their horse’s welfare, but also felt they may not always recognise deterioration resulting in poor QOL. Some participants found setting criteria with owners for when welfare declined to a certain point to re-evaluate treatment plans useful. Participants described that QOL during recovery and long-term were considered when deciding on treatment or euthanasia. They also discussed that colic surgery, for example, required a lot of aftercare and box rest and that some horses may not be able to cope with this. Where possible, participants would try and find compromises that could work for individual horses. Participants discussed the concept of horses being able to have a ‘good death’. This typically involved planning ahead for euthanasia and not waiting until the horse was already suffering from very poor welfare before deciding to euthanase. It was agreed that it was better to euthanase a horse a bit early rather than late, as this would prevent unnecessary suffering. Some participants framed euthanasia as a treatment option. Euthanasia was reportedly preferred over rehoming a horse that was very elderly or chronically ill, which would lead to an uncertain future and the potential for a life of poor welfare.

*[Telling owners] “You know it’s currently not in a really bad shape so I would rather do it now than when he’s suffering and can’t get up…”*

Rosewood, Veterinary Intern

The most commonly described cause of welfare problems by participants was owners failing to recognise that their horse was suffering. Participants reported that, in their experience, owners may ascribe changes in health to ‘old age’ and expect a certain level of decline. They felt this lack of recognition was especially likely in chronic conditions where there was a slow decline, which could mean owners were less likely to notice. Participants described that their role was to help owners understand that their horse was experiencing pain, as they would then be more open to treatment or considering euthanasia. Using formal QOL assessments was recognised as an option for assessing welfare over longer periods in chronic cases. Alternative methods also suggested included owners keeping a diary and taking videos of their horses to track changes over time.

*“Well, let’s do a pain relief trial and I want you to keep a diary”…that almost flicks the switch of “this is pain related”. So then when that horse does start to deteriorate, there’s then a recognition that rather than, “oh, he is looking old again,”, it’s, “he’s in pain again.”*

Poppy Ridge Charity, Veterinary Surgeon

However, participants reported that owners did not always agree their horse was in pain or was suffering, which made it challenging to engage them in discussions about treatment. Sometimes, they felt this was because owners had a very strong emotional attachment to their horses and did not want to acknowledge declining welfare, and so were reluctant to seek advice or discuss euthanasia. Alternatively, participants reported the experience of owners having misconceptions or preconceived ideas about certain treatments or diseases, which they felt could affect the likelihood they would engage in treatment or euthanasia discussions. This included misconceptions about the natural dying process for some owners, which was described by participants as potentially slow and painful in chronic cases.

*…no horse really, very, very rarely does a horse just die peacefully. It dies because it has spent all night trying to get to its feet. As a prey animal, it knows, if I can’t get to my feet, I’m somebody’s dinner, and it struggles and panics…*

High Oaks, Veterinary Surgeon 3

#### 3.2.2. Finances vs. Welfare

Participants felt that poor welfare could result when horse owners delayed making a decision due to financial concerns, for example, the cost of treatment or euthanasia and body disposal. It was noted that the cultural shift in the role and value of horses had led to euthanasia being expensive, whereas, in the past, horse owners may have been able to obtain money from taking their horses to an abattoir or knacker.

*…we spent a week trying to tell the owner that it needed to come in and she kept saying she had absolutely no money…and it got to an emergency on the Saturday where the horse was nigh on nearly dead. But she suddenly said, ‘I’ve got £10,000, please save my horse’, and the horse, the horse collapsed while trying to load it onto the box…*

Whitedale, Veterinary Surgeon 1

Insurance was also mentioned as a barrier to decision-making and welfare, both where owners did not have insurance coverage, or it did not cover the full cost of a treatment, for example, colic surgery.

*We see a lot of horses that need colic surgery, and either owners aren’t insured, or they’re insured and insurance is generally up to [£]5000, and nowadays we can’t do colic surgery for [£]5000.*

Whitedale, Veterinary Surgeon 1

Participants discussed the fact that some owners are reluctant to spend money on treating older or low-value horses. Some had experience with owners who would euthanase a horse with a treatable issue and then buy a new horse instead, which could be cheaper. This was described as distressing for participants. Poppy Ridge Charity participants discussed how, in certain contexts, they would fund a veterinary surgeon visit, medications, or euthanasia if they thought it was in the best interests of a horse and the owner (or loaner for charity-owned horses) could not afford or was not going to pay for it. These participants also discussed the difficulty that equine charities, in general, faced balancing resources, including the welfare of one individual horse needing treatment versus potentially many horses that could be helped for the same cost.

### 3.3. Relationships

**Figure 3:**
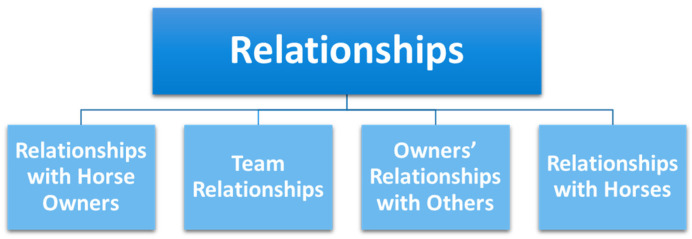
Diagram showing the theme ‘Relationships’ and associated subthemes.

Figure 3 displays the subthemes within the theme “Relationships”.

#### 3.3.1. Relationships with Horse Owners

Continuity of care was perceived by participants as being important for building rapport with owners, which could then lead to more successful decision-making. Introducing conversations about end-of-life gradually (for chronic cases where this was suitable) over several visits was often seen as preferable, as it allowed trust to build.

*It’s being able to build that relationship, so the client is confident in having that chat and maybe having a discussion about when the time is right…*

High Oaks, Veterinary Surgeon 1

Veterinary team members could have different roles and relationships with owners. Participants reported that owners sometimes feel more comfortable speaking to a nurse or other member of staff rather than the veterinary surgeon principally involved in the case, or want to obtain another opinion. Veterinary team participants reported aiming to be empathetic with owners facing end-of-life decisions, understanding that this would be upsetting and challenging for them. They felt this could also improve communication and their relationship with the owner. Some talked about it being their role to help owners process the loss of their horses. However, providing empathy could also be emotionally draining. Participants felt that they were sometimes subject to the stressful responses of owners who were upset during euthanasia decision-making. They acknowledged, though, that they were not aware of other difficulties that owners might be confronting at the time, which could be compounding their stress.

*…the next day she turned up with two bunches of flowers for both the vets…because she knew she’d been really abusive. But you know, often clients are really stressed, so we get a lot of the brunt of them being shitty with us…*

Whitedale, Veterinary Surgeon 1

#### 3.3.2. Team Relationships

Participants discussed finding their work emotionally challenging at times. Relationships among team members played an important role in mitigating this and were particularly discussed by participants from Poppy Ridge Charity and Whitedale. Whitedale participants emphasised the importance of being supportive to colleagues who were struggling and allowing team members to feel that they could raise issues they faced with other members of the practice.

*…there’s nothing more true than it’s okay not to be okay. And, you know, it’s important we do share that burden essentially.*

Whitedale, Veterinary Surgeon 5

Trust, strong relationships, and joint decision-making among charity staff were described as key to coming to mutually acceptable decisions and sharing the burden of responsibility. Having a permanent veterinary surgeon reportedly allowed trust to be built between them and other staff, improving communication. However, despite euthanasia decisions being shared, not all team members were involved in each decision. A hierarchy also existed where some staff had more decision-making power than others, and the most senior staff member had ultimate responsibility for the horses’ welfare and decisions. Grooms provided day-to-day care for horses and often had close relationships with them, described as being similar to that between a horse and its owner. However, they reportedly had less decision-making authority than senior staff. Participants recognised this and emphasised the importance of providing support and fully explaining decisions. When these participants had to make difficult decisions, for example, for less clear-cut cases or horses they were emotionally involved with, they found support from colleagues valuable.

*So, it’s easy for us to sit back and make the decisions and do it professionally. But actually, it’s the understanding that…it’s going to affect [the grooms] differently. And I think we’ve all learnt to really offer that support as well, that anyone can walk up to anyone and talk about it.*

Poppy Ridge Charity, Charity Staff Member 4

Some examples of poor communication and conflict were given. Within Poppy Ridge Charity, this was described as having occurred in the past between charity staff and a visiting veterinary surgeon but had reportedly not been an issue since the charity employed their own veterinary surgeon, who staff had built trust with.

*I think that’s where we’ve had problems in the past…it’s been where that decision hasn’t been sort of really communicated properly by, like you say, whoever the vet was at the time.*

Poppy Ridge Charity, Charity Staff Member 1

#### 3.3.3. Owners’ Relationships with Others

Participants gave examples of situations where the joint owners of horses disagreed about care and treatment decisions. They discussed the need to act carefully in these situations to avoid individuals taking opposing positions. Participants also discussed relationships between owners and others in the yard. These included where owners sought advice from yard owners who knew the horse well and circumstances when other yard members tried to become involved in decisions without being asked. The latter was described as complicating decision-making and increasing stress for owners and veterinary surgeons.

#### 3.3.4. Relationships with Horses

Participants reflected that the role horses played in owners’ lives could affect their relationship with them and the end-of-life decisions made. It also could potentially affect how participants approached end-of-life conversations with owners. They reported many owners viewed their horses as family members and perceived that these people would potentially struggle more to make euthanasia decisions. In contrast, professional horse owners (those who keep horses to earn money as part of a business) were perceived as likely to be more pragmatic and practical decision-makers, which could make management of the case easier for veterinary team members.

*If they were a little bit more of a professional, they’ll perhaps be more detached and more readily open to [euthanasia] as an option…*

Rosewood, Receptionist

However, owners having a strong relationship with their horses and knowing them well was described by participants as being valuable. They felt it meant owners were more likely to notice changes in their horses which may indicate health or welfare concerns.

Participants at Poppy Ridge Charity reported team members often built relationships with individual horses, especially long-term residents. This was the case with management and senior members, too, not just grooms. It was noted that, even if a case was clearcut in a veterinary sense, emotionally, the decision could be very difficult, and having support and input from team members was especially valued. If the relationship a member of staff had with a horse impacted their ability to make a decision about its end-of-life, then the decision would be escalated above them, or additional opinions would be sought.

*…I did that with Stormy, you know, I had to escalate it because I was too emotionally involved.*

Poppy Ridge Charity, Charity Staff Member 3

### 3.4. Decision-Making

**Figure 4:**
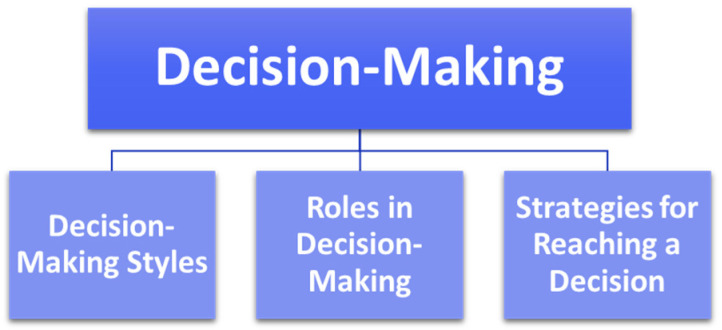
Diagram showing the theme ‘Decision-Making’ and associated subthemes.

Figure 4 displays the subthemes within the theme “Decision-Making”. 

#### 3.4.1. Decision-Making Styles

Participants discussed using several different decision-making styles depending on the context, specific situation, and individuals involved. Shared decision-making (SDM) was described when actively making decisions with owners and other significant people they wanted to involve, such as family members. Owners’ perspectives were described as valuable as they knew their horses well, often implemented treatment or aftercare, and were the ones with the legal responsibility of making the final call.

*…they know them so well and you’ve got to get them onside, and you’ve got to make that decision all together like the owner and vet.*

Whitedale, Veterinary Surgeon 6

Continuity of care was reportedly valued in this style of decision-making, as building trust between the owner and the veterinary team can encourage collaborative decisions. Participants described using different communication and decision-making styles in acute compared with chronic situations. The latter had a greater possibility of allowing SDM, as participants reported that conversations about end-of-life could be introduced gradually over several visits. Involving owners in ongoing QOL assessments was considered important in SDM, as was taking into account their personal circumstances and adapting aftercare or treatment to work for them and their horses. This could be the difference between owners choosing treatment or euthanasia.

Participants also reported steering decision-making or taking more responsibility for making decisions in some circumstances, which will be termed veterinary practitioner-directed decision-making. This was especially described in acute and emergency situations where the participants thought that the horse was suffering and needed treatment or euthanasia but felt the owner was struggling to come to a decision about which course to take. In these situations, participants described giving strong guidance and recommendations to owners. In some cases, owners reportedly actively sought direction from veterinary team members and asked what they should do.

*[In an emergency] People are usually quite distressed, emotional…and actually they need the vet to be making decisions and to give the owner very black and white decisions.*

Rosewood, Veterinary Surgeon 2

In some cases, descriptions from participants suggested either the veterinary surgeon or owner would try to shift the responsibility for the euthanasia decision to the other party. Sometimes, participants described shifting the burden of the decision away from owners onto themselves and the veterinary team.

*…it’s equally as important that we come in and take that burden off the owner, so that it’s less of a decision.*

Whitedale, Veterinary Surgeon 5

In other instances, veterinary professionals described shifting the responsibility to the owner to allow autonomous decision-making. This was discussed as being taught at veterinary school as the appropriate response, and the reason veterinary surgeons should not directly answer the question, “what would you do if it was your horse?”. Some participants declined to answer this question, while some were happy to answer, perceiving this as giving owners their clinical opinion. Others said their answer would be owner-dependent.

*You’re not telling them that that’s what they’ve got to do, but you’re giving them an honest opinion of what you think the future holds for it, what the options are for them and what your advice is as to which option they should take.*

Rosewood, Veterinary Surgeon 2

Participants also perceived that sometimes a referring veterinary surgeon would avoid taking responsibility for a euthanasia decision by shifting it to the referral hospital.

#### 3.4.2. Roles in Decision-Making

The different roles that horse owners and veterinary team members play in end-of-life decision-making were touched on during participants’ conversations. Horse owners’ roles were described as knowing their horse, providing information about its individual personality and behaviours, and providing day-to-day care. The veterinary surgeon was described as the main person having conversations and making decisions with the owner, including advising owners, bringing together multiple views, and managing difficult or stressful situations to allow an environment for decision-making. However, veterinary surgeons also emphasised that their role in decision-making was advisory. So, even when they took a more veterinary practitioner-directed approach or tried to shift the burden of decision responsibility towards themselves, owners always had the final choice on what to do for their horses.

*…we’re not there to police them, we’re there to advise them…we must never try to be dictatorial to clients, that’s not our role.*

High Oaks, Veterinary Surgeon 2

Nurses described their role as supporting the owner through the decision, with owners coming to them for additional advice or with questions. Participants perceived that sometimes owners would find it easier to talk to nurses. Whitedale participants reported that a lot of their nurses knew the practice’s clients, which likely helped owners feel comfortable having additional conversations with them. Examples of nurse involvement were given for horses that had been referred to hospitals, as opposed to ambulatory cases.

*…we’re there to support them through their decision…whether it be holding their horse because they can’t, or…they just want to talk to somebody different, so, you can be an ear to listen to if they’ve not got a supportive friend to phone.*

High Oaks, Nurse

The reception team were the first port of call when owners called up with an issue with their horses. They described that owners would sometimes call asking for advice and that their role was to help find solutions for them by taking information to pass onto veterinary surgeons and organising a veterinary surgeon to attend so they could advise the owner. They also discussed talking distressed owners through what was going to happen and the importance of reassuring them and keeping them calm. Whitedale receptionists had developed a script that could be used for discussions about euthanasia to ensure all owners receive similar information.

*…just making sure that they have confidence in you that we’re going to get someone there to them. And just making sure that you end the phone call in a calm way, and making sure that they know that their horse is going to get the best care.*

Whitedale, Receptionist

Reception team participants gave examples of how showing empathy to owners could improve communication when discussing euthanasia arrangements and reassure them they were not approaching the situation with a purely business mindset. These participants also described showing discretion in how they asked for payment from owners for euthanasia. They recognised it could be upsetting and gave examples of giving owners the option of paying in advance or after. Some described sometimes delaying sending the invoice, so owners did not receive this straight after euthanasia.

*…we tend to monitor when in the month horses are put to sleep, and we then decide when we’re going to send the invoice.*

High Oaks, Office Manager

The reception team also provided information about services available and costs, helping owners decide their preferred body collection and disposal options.

#### 3.4.3. Strategies for Reaching a Decision

Participants reported employing different strategies to help encourage or speed up decision-making where they felt this was needed. One method was to be proactive by discussing costs early and managing owner expectations of treatment outcomes and the euthanasia process. Whitedale reported holding colic evenings where they encouraged owners to plan for a colic emergency to prevent decision-making delays should this occur. Plans consisted of what they would do, including transport and insurance cover.

Another method participants described was using persuasive techniques, such as presenting black-and-white options to owners, especially in emergencies, and being blunt to emphasise the need to take action. Participants also reported using the results of clinical tests and pain medication trials to demonstrate to the owner that they believed the horse was suffering and that some form of action should be taken. Additionally, they described emphasising welfare concerns and giving examples of potential negative outcomes if the horse did not receive treatment or euthanasia.

Veterinary surgeons also gave examples of managing the decision-making environment, such as by taking owners away from crowds of other yard members so they could have a private conversation. Taking the owner away from their horse was another strategy reportedly employed when participants wanted to have important conversations with the owner’s full focus.

Another strategy mentioned in all focus groups was “planting the seed”. This meant putting the idea in the owner’s mind that their horse’s welfare was either poor or declining. This was used in chronic cases to gradually introduce the idea, allowing owners to reflect and discuss with the veterinary surgeon again in follow-up visits. According to participants, this approach allowed trust and a relationship to be built between veterinary surgeon and owner.

*If they’re completely blind to it, you don’t want to go straight in for something they’re not expecting. But you just want to…sort of plant a seed that actually things aren’t quite right, and hopefully they can then come to the conclusion themselves, with a bit of assistance.*

Rosewood, Veterinary Intern

Resources were reportedly sometimes used to assist decision-making. For example, guidelines created by the British Equine Veterinary Association to prompt and guide conversations about consent and insurance. QOL tools and scores were discussed, which could be used to help owners assess their horse’s welfare and track changes, but this was not commonplace. One participant also brought up the fact that it could be useful to direct owners to bereavement resources, such as the BHS Friends at the End scheme [23] or Blue Cross pet loss service [24]. However, this was not currently being performed at their practice. Some participants thought additional resources to prompt end-of-life discussions and guide decisions would be beneficial, especially for less experienced veterinary surgeons. However, others were doubtful that having something like this could be adaptable enough to suit the varying situations they encountered in practice.

*…I’ve never had to make the decision or advise the owner about making the decision yet…I think I would find that quite a hard conversation to steer and not pressure the owner, but give firm guidance, if that make sense. So yeah, if there was something…that prompted a conversation between the two that might be quite helpful, I think.*

Whitedale, Veterinary Surgeon 4

### 3.5. Emotional Toll and Coping Strategies

**Figure 5:**
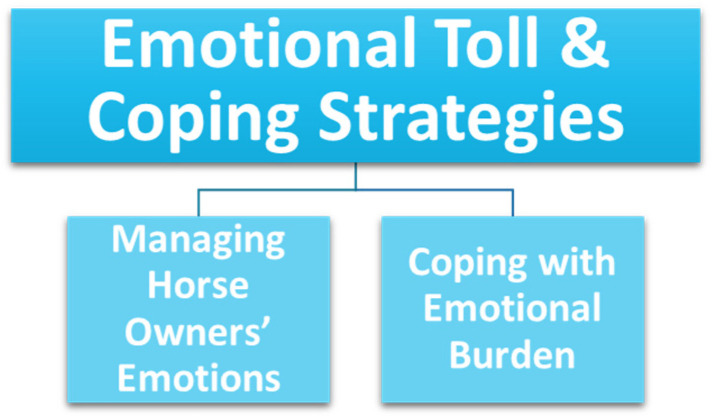
Diagram showing the theme ‘Emotional Toll and Coping Strategies’ and associated subthemes.

Figure 5 displays the subthemes within the theme “Emotional Toll and Coping Strategies”.

#### 3.5.1. Managing Horse Owners’ Emotions

Participants discussed how making end-of-life decisions was stressful and upsetting for owners, including seeing their horses struggle and coming to terms with the fact their horses would require euthanasia soon. Regret and guilt were discussed, for example, owners regretting leaving it too long to treat or euthanase their horse and feeling guilty about letting welfare decline or guilt resulting from emergencies. Participants discussed that owners’ guilt could also result just from choosing euthanasia or from being unable to afford treatment for their horses. It was seen as important to be non-judgemental and not to pressure owners into a decision they were not comfortable with. Participants addressed how euthanasing their horses could be a relief for some owners, especially for elderly horses with health problems requiring a high level of care.

*As a professional person you should allow them to accept that release. You shouldn’t allow them at all to feel any guilt about their decision…and allow them to feel that it’s the right decision, because the vet agreed.*

High Oaks, Veterinary Surgeon 3

Participants reported instances of reassuring owners that they were doing the right thing by choosing euthanasia, which they felt could help prevent or alleviate owners’ guilt. In some cases, they reported doing this regardless of whether they believed it, for the owner’s benefit.

*…if I really think that that was the right decision, I’ll tell them after I’ve done it…you know, “for what it’s worth, I think you made the right decision. I would have done the same thing”. I often would say it regardless of what I would’ve done. But quite a few have phoned afterwards to say that they appreciated me saying that.*

Rosewood, Veterinary Surgeon 1

Some veterinary surgeons described encountering owners seeking affirmation when euthanasia may not have been necessary, and the veterinary surgeon did not agree with the decision, which put them in an uncomfortable position.

*…[the owner is] trying to make you say, “no, it’s okay, it’s the right thing to do”…I used to, when I was younger, try and sort of appease that. But I find it harder and harder to do now because it’s so upsetting for me putting down a horse that I know I can fix.*

Whitedale, Veterinary Surgeon 1

Participants shared some examples of what they perceived to be an abnormal or inappropriately exaggerated display of emotion from owners. They described finding these experiences stressful and difficult to navigate.

*…she thought she was going to sleep with it until it was collected the next day. She brought clothing and laid it all over the horse…and a friend was videoing her lying next to it dead on the floor…she was dramatic vomiting outside, she was in a right mess…It was horrendous, but then you don’t want to be rude to them…But then what, what do you do in that situation?*

Rosewood, Nurse

Shock and other strong emotions were sometimes viewed as hindering decision-making. Emergency situations were usually more stressful than chronic cases, often for both the veterinary team and owner, as people were dealing with shock and were unlikely to have been practically and emotionally prepared.

*I think there’s a difference between when it’s their old horse or whatever that they’ve decided he’s got to go. I think they, emotionally they’re in a probably stronger place than when it’s, “oh my God, it’s colic, it’s going to die, it’s broken it’s leg, or it’s doing this”, because then that’s like they’ve got the shock value on it.*

Rosewood, Receptionist

The impact of having an emergency and/or end-of-life plan in these situations was discussed as being positive. Some participants felt that making plans and decisions in advance could help practical aspects of euthanasia happen smoothly and make the experience less upsetting for the owner.

*…it’s why planning these horses’ ultimate euthanasia is actually a really good thing because…you can actually do things like have the collection arranged beforehand so that all goes smoothly…*

High Oaks, Veterinary Surgeon 2

Participants described that owners could find it upsetting if they could not afford their preferred method of body care. Participants recognised that individual cremation with ashes returned, which owners often wanted, was expensive. Some reported supporting owners to find alternative ways to create a memorial to their horse, without this expense.

*…the people that haven’t got the money to do it, like you can kind of say, “you know, we could do it like a nice memory board of some pictures and things and have some mane and tail”.*

Rosewood, Nurse

#### 3.5.2. Coping with Emotional Burden

As discussed in ‘Relationships’, support from colleagues was important in mitigating stressful and emotionally draining aspects of participants’ roles. Other strategies used are discussed here. Participants at Whitedale advised not taking it to heart when owners behaved abusively, as often it resulted from a culmination of stresses. The use of alcohol was also jokingly referenced at Whitedale as a coping method for work-related stressors, but participants recognised that mental health was a serious issue affecting veterinary professionals.

*…ultimately there could be other traumas or ordeals that these clients [are] going through…And you often end up being a punching bag because their emotions are already running high…definitely thick skin, shake it off.*

Whitedale, Veterinary Surgeon 5

As mentioned in ‘Finances vs. Welfare’, participants found being asked to euthanase horses with a treatable condition upsetting, as well as encountering horses that they believed were suffering, but owners did not agree. Participants also described that carrying out multiple euthanasias in a short space of time had the potential to cause distress, as did anxiety that they had pushed owners to euthanase their horses too early. This was a concern, especially among less experienced veterinary surgeons. Some participants reported reconciling euthanasing horses when they deemed it unnecessary for their welfare by emphasising that this was never a wrong decision. They felt this way as it would prevent rehoming multiple times and potential poor care and suffering in the horse’s future. Participants acknowledged that dealing with death or other stressors in their personal lives could compound with stress from their work and impact mental health.

Euthanasia was framed as a positive in some cases, and described as “rewarding”, as it could resolve welfare issues for horses and also sometimes provide relief for clients. Participants commented that they were glad they were able to offer euthanasia for their patients and compared being unable to do this for human family members who were suffering.

*…I find [euthanasia] one of the most rewarding parts of my job actually, because it does resolve welfare problems, it resolves problems for clients, and you know, you see how people suffer in end-of-life…*

High Oaks, Veterinary Surgeon 2

## 4. Discussion

This study explored in depth the experiences and roles in end-of-life and euthanasia decisions among a range of equine veterinary and charity team members. Four major interrelated themes were generated, ‘equine welfare’, ‘relationships’, ‘decision-making’, and ‘emotional toll and coping strategies’. Key issues featuring within multiple themes related to communication, finances, and chronic vs. acute cases. Patient welfare was identified as the participants’ priority when making decisions. However, horse owners did not recognise when welfare declines were described as a potential barrier to making end-of-life decisions. Chronic cases reportedly allowed more time for relationships between the owner and veterinary team to be built and so provided greater opportunity for shared decision-making (SDM) than in emergencies. Different approaches to decision-making were favoured in different contexts. Participants found aspects of their role emotionally challenging, but the impact of these could be mitigated through open communication and strong relationships with colleagues. Although end-of-life decisions could be difficult and stressful, euthanasia could also be a rewarding aspect of practice for participants when resolving equine welfare issues.

Building relationships with owners and continuity of care were perceived as important for successful communication and decision-making. This has been recognised in both human and veterinary medicine and can improve trust between healthcare providers and patients or owners/caregivers [25,26]. Sharing experiences such as decision-making and grief with others has been found to be a stabilising tool for caregivers of human relatives [27] and may also apply to companion animal caregivers. The vet–owner relationship is influential in colic treatment decision-making, with owners feeling additional security when a known veterinary surgeon is in attendance [25]. However, this may not always be possible, for example, in an emergency [28]. Although there would typically be a main veterinary surgeon involved in decision-making with the owner, participants commented that some owners felt more comfortable asking advice from nurses or other team members as well, similar to findings in human and companion animal medicine [12,26]. A team approach to equine end-of-life care and euthanasia decision-making can be beneficial for owners, especially if their horse has received care from multiple people. Horse owners may speak to multiple members of the veterinary team as a way of canvassing opinions on whether they should opt for euthanasia or a specific treatment, as reported in human healthcare contexts [29].

Participants perceived that, if owners knew their horses well, they were more likely to recognise changes in health and welfare. They described valuing owners’ understanding of their horses, as this perspective could influence the best course of action for a horse needing treatment or euthanasia. Owners can also become more aware of and better able to interpret their companion animal’s QOL over time [9], which is valuable for decision-making. Research has found horse owners interpreted the severity of colic based on their connection with their horse, which impacted when they sought veterinary advice [25]. However, owners may not always recognise declining welfare [2,30], and some interpret changes in their horses depending on how these impact their horse–human relationship [31]. It is common for UK leisure horses to be kept at livery yards, and yard managers have a strong influence on owners’ management practices and care decisions for their horses [32]. Participants in this study described how yard managers could often be helpful, as they were trusted by owners and also knew the horses in their yard well.

Participants emphasised that, during all end-of-life decision-making scenarios, their priority was the individual horse’s welfare. Although they believed that, in the vast majority of cases, this was also true for owners, they also considered that owner misconceptions or preconceived ideas could contribute to poor decision-making and negatively impact equine welfare. For example, participants encountered owners who reportedly wanted their aged horses to die a peaceful, natural death despite the fact that the majority of horses will require euthanasia rather than dying naturally [33,34]. This desire could potentially delay owners in seeking veterinary advice. Participants viewed part of their role as addressing owner misconceptions and educating them on the disease trajectory and prognosis so they could make fully informed decisions. However, in some cases, they reported that owners did not agree with their assessment that their horse’s QOL was poor, which has been described as a challenge in other studies [35]. Research has found that horse owner opinions on welfare can be subjective and contextual, with differing views on the point at which a horse’s QOL might be compromised to the point where intervention or euthanasia is required [36]. Owners and veterinary professionals may also have different perspectives on what good equine welfare looks like, which could lead to different priorities when making treatment and end-of-life care decisions. It would be beneficial for veterinary surgeons to discuss with owners their understanding of their horse’s welfare, how they evaluate this, and what their priorities are, including sharing their own perspectives.

Financial concerns were reported as barriers to timely advice-seeking and decision-making, restricting treatment options, and negatively affecting equine welfare, similar to the findings of other companion animal studies [8]. Veterinary team members found financial barriers frustrating. Previous research has found horse owners usually say that although there is some financial influence, welfare rather than finances is the main factor in treatment decisions [37]. In contrast, participants in this study felt it was often a significant barrier. The availability of insurance was perceived to contribute, as equine insurance in the UK commonly does not cover the full cost of some treatments, such as colic [38].

Equine charity participants discussed how the charity sector is faced with the difficult task of balancing finances and resources with equine welfare. Many charities are at capacity and so focus on rescuing and rehoming serious welfare cases and generally cannot take in horses that owners can no longer care for or do not want [14]. Anecdotally, charities also tend to promote the idea that euthanasia is “better a week too soon than a day too late” [39]. Participants discussed the concept of providing a good death for horses. One aspect of this was encompassed by this phrase, emphasising that it was preferable to euthanase horses before their welfare declines to a point at which they are severely suffering. Some participants framed euthanasia as a treatment option and a positive choice for welfare, which is a concept promoted by several equine organisations [39,40]. This has been suggested in wider companion animal research [41]. Framing euthanasia in this way may influence it to be viewed as a more acceptable choice to owners and veterinary practitioners, as opposed to just “giving up” on treatment.

Some participants described euthanasia as rewarding as it could alleviate suffering for both horses and humans, as described in a qualitative companion animal study [42]. Despite some positive framing of euthanasia, participants also gave examples of team members who struggled with feeling that maybe they had pushed owners towards euthanasia too soon. Many veterinary surgeons find euthanasia difficult; according to a systematic review, it can be one of the aspects of their job that leads to poor mental health, especially when newly qualified [43].

A strong emotional response from owners was sometimes perceived by participants to make decision-making more difficult, which is also reflected in other equine research [35]. Similar perspectives have been reported from studies of companion animal veterinary surgeons who aimed to manage owners’ emotions to facilitate what they felt was rational decision-making [8]. In other studies, some horse owners have described how they felt their attachment to their horse had negatively affected their ability to make decisions about end-of-life [5] and assess QOL [35]. Poppy Ridge Charity participants also described instances where they felt their relationship with a particular horse had impacted their ability to contribute to a euthanasia decision. In these situations, they especially valued input from other team members to come to a joint decision. Participants considered that professional horse owners were more practical and less emotional and struggled less with euthanasia decisions compared with leisure owners. However, survey and interview data demonstrated that both these groups formed strong relationships with their horses and experienced grief and emotional burdens [5]. Therefore, veterinary teams should not assume professional owners are less in need of support during end-of-life decision-making.

Participants understood that euthanasia decisions are very difficult for owners and aimed to provide empathy to owners but could also find this draining. These findings are similar to those in companion animal studies, where navigating decision-making was typically perceived as more stressful than carrying out euthanasia [42]. This was due to the pressure to support owners’ emotional needs and having to balance this with the animal’s often declining welfare [42]. Participants sometimes took on the role of counsellor and also encountered owners who behaved aggressively or abusively towards them, adding additional burden. Difficult relationships with owners such as this can contribute to poor mental health in veterinary professionals [43]. Strong relationships among participants and work colleagues were important to cope with difficult interactions and moral stressors surrounding euthanasia, which has also been identified in other veterinary research [44]. Having supportive co-workers has been found to reduce feelings of burnout and suicidal ideation in veterinary surgeons, whereas alcohol consumption as a coping strategy exacerbated these [45]. A structured review of publications relating to veterinary suicide found that veterinary surgeons in the UK are approximately twice as likely to die from suicide as human health care professionals [46]. Mental health within the sector warrants further research which includes all members of the veterinary team.

The preferred decision-making approach varied depending on the context. One strategy described in chronic cases was “planting the seed”, which was used to build up to decisions over time whilst fostering trust with owners and allowing them time to reflect on available options. This approach has been used in human medical situations, for example, by physicians of patients with chronic diagnoses [47]. SDM was also valued in chronic cases, where participants again discussed the benefit of continuity of care and building relationships. Although no participant explicitly used the term SDM, they described approaches such as making decisions “together” or making a “team decision”. Studies in small animal practice have found that SDM was favoured by most owners (65%) [48] and veterinary surgeons (86%) [49]. Horse owners in other studies have also reported valuing their veterinary surgeon as a partner who contributes complementary knowledge to their own for decision-making [35]. Families of human palliative care patients found the responsibility for end-of-life care decisions a high emotional burden related to increased depression and grief [50]. The ability to share and so lessen the decisional burden may be a protecting factor for mental health in both charity staff and veterinary teams and owners.

However, SDM is not always the most preferred or appropriate model in both human and veterinary medicine. In the studies referenced above, approximately 14% of veterinary surgeons [49], and 25% of companion animal owners [48], preferred a paternalistic or veterinary practitioner-directed approach. Again, there are similar findings from human medicine [51]. Preferences for decision-making are likely to vary depending on several factors, including the age and experience of those involved and the specific scenario. These differing preferences highlight the importance of tailoring approaches to the individual situation and owner. Participants in this study were more likely to describe directing decision-making in emergencies or where they felt that an owner was struggling to make a decision on their preferred course of action and the horse was suffering. Where SDM is preferred, important aspects such as building a relationship with the owner may not be possible in an emergency [28]. However, others, such as sharing information and goals, should still be possible when under a time-pressure situation. Creating an emergency plan could allow decisions to be shared and made in advance and may be able to reduce the stress felt in emergencies.

In some cases, participants emphasised that their role was to educate and advise but that the owner had the responsibility for making a decision. This contrasted with the examples given of more paternalistic approaches, where participants gave more direct guidance on the path they thought owners should take. The autonomous approach to decision-making aims to empower patients, but it can also be interpreted as health care providers abdicating responsibility for decisions and may not be aligned with patients’ wishes [52]. Although it is important to allow owners to make decisions without feeling they are being pressured [53], they may not want to take a fully autonomous role in decision-making (preferred by only 10% of owners surveyed) [48]. This aversion to a fully autonomous approach has also been observed in human healthcare studies [51]. Eliciting owners’ desired level of involvement in decision-making was one of the decision components identified in a scoping review of veterinary decision-making models [54]. This is likely to be beneficial in building relationships and increasing owner satisfaction. Components central to SDM, such as knowledge exchange between the horse owner and veterinary team, could still be enacted even if the owner prefers to take the lead in decision-making or would rather the veterinary team assume this role instead of engaging in a full joint decision.

When owners shift the responsibility of decision-making towards the veterinary team, they may be seeking an expert opinion as they feel they lack the knowledge to make a decision themselves [55]. Another potential reason may be that they are trying to avoid or alleviate feelings of guilt, which are commonly experienced during euthanasia decisions [8]. Participants recognised this and reported making attempts to prevent or alleviate owners’ guilt, which veterinary practitioners in other studies have also described [8,25]. One way this was achieved was by reassuring owners that they had made the right decision by choosing euthanasia, which owners reportedly appreciated. Small animal owners in one study reported that providing affirmation that euthanasia was the right decision was the most important way their veterinary practitioner could give them emotional support [6].

Examples described in this study suggest that, sometimes, participants handled decisions with owners well, but, at other times, they struggled with these, and approaches could be improved. This highlights the difficulty in achieving ‘ideal care’ in complex and changing real-world settings, which has been described in human healthcare [56].

Several resources providing education and support for different aspects of end-of-life exist, developed by equine charities and organisations [23,24,39,57]. Some participants felt that signposting owners to these could be beneficial, as they may not be aware of their existence otherwise [25]. However, participants reported that, in practice, this was rarely performed, and it is not currently clear how widely such resources are being used in UK veterinary practices. Early-career veterinary surgeons are at greater risk of compassion fatigue and poor mental health [7,43], so it may be that resources to support communication and decision-making are most beneficial to this group.

There are some potential limitations associated with this study. Participants may have felt pressure to agree with senior members during focus groups. Veterinary surgeons made up the largest group of participants, so their views may be more represented than those of other team members. Those with a greater interest and experience in end-of-life decisions may have been more likely to participate. The sample gathered for this study was opportunistic and aimed to explore and understand some of the views and opinions of professionals involved in equine end-of-life decision-making. Therefore, the results may not be representative of the wider population of UK equine charity and veterinary teams. All focus groups were single centres, reducing the number of establishments represented. However, this was chosen to increase the likelihood of participants having rapport with each other and sharing knowledge of the processes and culture at their establishment.

## 5. Conclusions

This study has provided insight into approaches taken to end-of-life decision-making by equine veterinary and charity teams and the challenges they face in this context. Veterinary teams aimed to provide continuity of care and build trust with owners, believing this improved decision-making. However, this was not always possible, and participants experienced difficult relationships and decision-making processes with some owners, which were stressful and negatively impacted their mental health. Financial limitations commonly complicate decision-making and increase emotional burden. Involving more members of the veterinary team in end-of-life care and euthanasia shared decision-making in certain scenarios could benefit both owners and veterinary teams. Charity teams found this helped share the burden of responsibility and contributed a range of expertise and knowledge to decisions. Varying decision-making styles were applied depending on the situation. While shared decision-making was valued in chronic cases, in highly emotional and emergency situations, participants were more likely to describe veterinary practitioner-directed decision-making. Difficulties navigating owner autonomy when directly asked for guidance were also discussed. Having a discussion with owners about their preferred role in decision-making would be valuable when approaching difficult decisions. The development of resources to be used by veterinary teams and owners to encourage planning for end-of-life care, support decision-making, and prompt difficult discussions would be beneficial. These should be adequately flexible to be applied to individual situations.

## Figures and Tables

**Table 1:** Number of participants in different roles in each focus group.

Participant Role	Practice or Charity Name			
	Rosewood	High Oaks	Whitedale	Poppy Ridge Charity
Veterinary Surgeon	2	3	6	1
Intern Veterinary Surgeon	1	1		
Veterinary Nurse	1	1	1	
Reception or Office Staff	1	1	1	
Charity Staff				6

## Data Availability

Anonymised data are only available on reasonable request from Amelia Cameron (amelia.cameron1@nottingham.ac.uk) due to confidentiality and privacy obligations to the participants.

## Supplementary Materials

The following supporting information can be downloaded at: https://www.mdpi.com/article/10.3390/ani15050678/s1, File S1: Interview Schedules for Equine Veterinary Practice and Charity Focus Groups; File S2: Vignettes for Focus Groups; File S3: Primary Researcher Personal Reflexivity Statement.
