# Supplementary material for: Experiences of End-of-Life Decision-Making in Equine Veterinary and Charity Teams
Source: Animals (Basel). 2025 Feb 26;15(5):678. doi: 10.3390/ani15050678 (PMC11898496; doi:10.3390/ani15050678)
Supplement: Supplementary file 1 [file animals-15-00678-s001.zip › Supplementary Material S1 - Interview Schedules.pdf]

Supplementary Material S1: Interview Schedules for Equine Veterinary Practice and Charity Focus Groups

Equine Veterinary Practice Schedule

1. To start with, please could everyone introduce themselves, and tell me a bit about their role within the practice?

   Next I'd like to share some example cases for you to have a look at, that I'll then ask some questions about.

2. To start with, please just look at Case 1 – Acute colic
   - How do you think the veterinary hospital staff should approach this situation?
   - Should the staff try to sway the owner's decision-making?
   - Are different members of staff likely be involved in different ways in supporting the owner and ensuring a suitable outcome for the welfare of the horse?

3. Next, please have a look at Case 2 – Elderly, lame horse
   - How do you think the attending vet and the practice should approach this situation?
   - What is the best way for the attending vet to bring up their concerns with the family?
   - Should the attending vet suggest euthanasia as an option?
   - If the attending vet concludes the horse's QOL is poor and euthanasia is the only humane option, but the owners still do not want to euthanase, how should the vet and the practice proceed?
   - How could other members of the veterinary practice team play a role in supporting the attending vet if necessary?
   - Is there any kind of support that can be offered to the owners?

4. Does your veterinary practice have a euthanasia or end-of-life protocol that you follow?

   If Yes:

- What does this involve/what aspects of euthanasia does this address (from decision making, to the procedure, to aftercare)?
- Is this something that's written and shared explicitly?
- How do you find using it?
- What are the pros and cons of this protocol?
- Are different roles defined for different members of staff?
- Would you like to see anything changed in the protocol?

If No:

- How do you decide how to proceed with a euthanasia case?
- Do you think it would be useful to have an EOL/euthanasia protocol, and if so, what would you like to see covered?
- Do different members of staff take on different roles?

Other:

- Is there anything relating to euthanasia or EOL care you think your practice does especially well?
- Is there anything you think your practice could improve on?
- Do you have any form of staff debriefing after a euthanasia or death has occurred?

Bonus Questions (5-8, if we have extra time left)

5. Next, I want to ask whether you have been involved in a specific euthanasia case that went especially smoothly?
   - What reason do you think this case went especially well?
   - Are there any other factors you think contribute to euthanasia cases in general going well?

6. Conversely, can you think of a time you have been involved in a specific euthanasia case that was difficult?
   - What factors do you think contributed to this case being difficult?

- Would you do anything differently if confronted with a similar case in the future?

7. Do you often get asked by clients, ‘what would you do if it was your horse’?
    - How do you answer this question?
    - Is this something you’re taught not to answer?
    - What do you think clients are looking for when they ask this?

8. Do you ever get asked to euthanase healthy horses?
    - How do you feel about this?
    - Do you ever attempt to change the owner’s mind?
    - Have you ever refused to euthanase a horse?

9. If you could, is there any advice you’d give your former self about dealing with euthanasia cases within your role? **(approx. 8 mins)**
    - Is there anything you wish you’d known when you first began your role?
    - Did you feel prepared for the first euthanasia case you dealt with?
    - Has your approach towards euthanasia cases changed at all?
    - Is there any advice you give to new employees?
    - What aspects of euthanasia cases do you think might be most daunting to newly qualified members of staff?

10. Are there any kind of resources on euthanasia decision-making that you think would be helpful if they were developed?
    - What would be useful to include/what kind format would be most useful?
    - Are there any resources or guidelines you have used previously?
    - In what contexts would such resources most likely be useful?
    - Are there any circumstances where such resources would be unlikely to work well?

Ask if hasn't already been covered/clarified in previous discussions

11. Before we end, I just want to clarify what each of your roles during a euthanasia case is, so x, yours is…? *(Ask each person)*

    OR (if unsure of different roles)

    Before we end, please could each of you clarify what your role is during a euthanasia case, starting with x?

12. Are there any final comments you'd like to add, or anything I haven't covered that you think is an important aspect of the euthanasia/EOL process?

Equine Charity Schedule

1. To start with, please could everyone introduce themselves, and tell me a bit about their role within the charity?

Next I'd like to share some example cases for you to have a look at, that I'll then ask some questions about.

2. To start with, please just look at Case 1 – Acute colic
   - How do you think the charity staff should approach this situation?
   - Do you have a policy here for colic cases? And for other diseases/injuries?
   - Would whether the horse could be rehomed, and whether it could go to a ridden home, affect the decision?
   - What other factors would influence the decision?
   - Who would likely be involved in making this kind of decision?

3. Next, please have a look at Case 2 – Elderly, lame horse
   - How do you think the attending staff member and the charity should approach this situation?
   - What is the best way for the staff member to bring up their concerns with the family?
   - Should the staff member bring up the topic of euthanasia?
   - If one of your horses out on loan had been seen by a vet recently, would you contact a vet to visit, or would this be left with the loaner?
   - How could other members of the charity play a role in supporting the attending staff member if necessary?
   - Is there any kind of support that can be offered to the fosterers?

4. Do you have a euthanasia or end-of-life protocol that you follow?

   If Yes:

- What does this involve/what aspects of euthanasia does this address (from decision making, to the procedure, to aftercare)?
- Is this something that's written and shared explicitly?
- Are there different protocols for horses kept onsite and those out on loan?
- How do you find using it?
- What are the pros and cons of this protocol?
- Are different roles defined for different members of staff?
- Would you like to see anything changed in the protocol?

If No:

- How do you decide how to proceed with a euthanasia case?
- Do you think it would be useful to have an EOL/euthanasia protocol, and if so, what would you like to see covered?
- Do different members of staff take on different roles?

Other:

- Is there anything relating to euthanasia or EOL care you think the charity does especially well?
- Is there anything you think could be improved on?
- How to you resolve disagreements between staff members involved in a decision?
- Do you have any form of staff debriefing after a euthanasia or death has occurred?

Bonus Questions (5-6, if we have extra time left)

5. Next, I want to ask whether you have been involved in a specific euthanasia case that went especially smoothly?
    - What reason do you think this case went especially well?
    - Are there any other factors you think contribute to euthanasia cases in general going well?

6. Conversely, can you think of a time you have been involved in a specific euthanasia case that was difficult?
    - What factors do you think contributed to this case being difficult?
    - Would you do anything differently if confronted with a similar case in the future?

7. If you could, is there any advice you'd give your former self about dealing with euthanasia cases within your role?
    - Is there anything you wish you'd known when you first began your role?
    - Did you feel prepared for the first euthanasia case you dealt with?
    - Has your approach towards euthanasia cases changed at all?
    - Is there any advice you give to new employees?
    - What aspects of euthanasia cases do you think might be most daunting to new members of staff?

8. Are there any kind of resources on euthanasia decision-making that you think would be helpful if they were developed?
    - What would be useful to include/what kind format would be most useful?
    - Are there any resources or guidelines you have used previously?
    - In what contexts would such resources most likely be useful?
    - Are there any circumstances where such resources would be unlikely to work well?

Ask if hasn't already been covered/clarified in previous discussions

9. Before we end, I just want to clarify what each of your roles during a euthanasia case is, so x, yours is…? *(Ask each person)*

    OR (if unsure of different roles)

    Before we end, please could each of you clarify what your role is during a euthanasia case, starting with x?

10. Are there any final comments you'd like to add, or anything I haven't covered that you think is an important aspect of the euthanasia/EOL process?
