# Supplementary material for: Experiences of End-of-Life Decision-Making in Equine Veterinary and Charity Teams
Source: Animals (Basel). 2025 Feb 26;15(5):678. doi: 10.3390/ani15050678 (PMC11898496; doi:10.3390/ani15050678)
Supplement: Supplementary file 1 [file animals-15-00678-s001.zip › Supplementary Material S2 - Vignettes for Focus Groups.pdf]

Supplementary Material S2: Vignettes for Focus Groups

Equine Veterinary Practice Vignettes

Case 1

A veterinary referral hospital is managing a case of acute colic. The horse has deteriorated since it arrived at the hospital and now clearly needs surgery. The horse is insured but the owner does not want it to go for surgery, as their previous horse had surgery and then had multiple episodes of colic afterwards before being euthanased.

The insurance company will pay for treatment (surgery), but because the condition is treatable the humane destruction guidelines of the insurance company will not allow payment for euthanasia or disposal costs. The insurance company would pay out if the horse deteriorates to such a point that euthanasia is recommended by the referral hospital instead of surgery.

The owner wants to euthanase the horse rather than send it for surgery, but is concerned about the lack of payment if they do so despite insuring their horse, and being able to manage the ensuing euthanasia and disposal costs.

Case 2

A veterinary practice has been asked to come out and attend a horse with a cut. The horse is elderly and has been seen over the years by the practice, but not recently in the last few years. When a vet arrives, the cut is fairly minor and can be treated easily at a low cost to the owners. However, the vet sees the horse is lame at a walk and can only move very slowly. He is arthritic and has suffered from laminitis in the past. The owners are a family and are all very attached to the horse, who has taught all the children to ride but is now retired, living in a paddock with their new younger horse.

The vet mentions their concern over the horse's lameness. The parents say the horse is just old but seems happy, and is doing better since they started feeding him turmeric. They also say the horse is still able to get up and lie down, but the teenage daughter says she has seen the horse struggling to get up after rolling and tried to help it, and that sometimes it gets chased by the younger horse. The

parents say the horse is happy and so deserves to die peacefully in it's sleep, and they also do not want to upset the children, especially the younger ones.

Equine Charity Vignettes

Case 1

An equine charity staff member finds one of the horses living onsite in the field with acute colic. One of the charity's vets assesses the horse, and ascertains that it either needs to go for surgery or to be euthanased.

The horse is not old and was previously healthy, never having experienced colic before. It has a calm temperament and had just been advertised for fostering as a companion that could also be worked in hand. Due to conformation issues, it is not suitable for a ridden home, but these do not impact on its physical health as long as it is not ridden. There is some concern that the horse has not had much experience of being stabled for long periods, especially individually, which could be required for recovering from surgery. The charity is currently at maximum capacity. The horse's main carer wants the horse to be sent for surgery, while the yard manager thinks the best option is to euthanase the horse now.

Case 2

An equine charity staff member goes for an annual visit to one of the charity's horses that is at a long-term foster home. The horse is elderly, and its health appears to have declined significantly since the last annual visit according to the notes previously made. The staff member sees the horse is lame at a walk and can only move very slowly. The horse is arthritic and has suffered from laminitis in the past. The foster carers are a family and are all very attached to the horse, who has taught all the children to ride but is now retired, living in a paddock with their new younger horse who has not come from the charity.

The staff member mentions their concern over the horse’s lameness. The foster carers say the horse is just old but seems happy, is doing better since they started feeding him turmeric, and that they do not see a marked difference since the last annual visit. They also say the horse is still able to get up and lie down, but the teenage daughter says she has seen the horse struggling to get up after rolling and tried to help it, and that sometimes it gets chased by the younger horse. Her parents say the horse is happy and so deserves to die peacefully in its sleep, and they want to continue caring for it until this point. The parents also do not want to upset the children, especially the younger ones.
