# Supplementary material for: Experiences of End-of-Life Decision-Making in Equine Veterinary and Charity Teams
Source: Animals (Basel). 2025 Feb 26;15(5):678. doi: 10.3390/ani15050678 (PMC11898496; doi:10.3390/ani15050678)
Supplement: Supplementary file 1 [file animals-15-00678-s001.zip › Supplementary Material S3 - Reflexivity statement.pdf]

Supplementary Material S3: Primary Researcher Personal Reflexivity Statement

I am a white, female postdoctoral researcher at the University of Nottingham. The research in this paper was conducted as part of my PhD, which I completed at the University of Nottingham. Alongside my research I help with teaching in undergraduate veterinary student equine practicals. I grew up in Surrey, and attended the University of Bristol for a BSc in Animal Behaviour and Welfare Science, and an MScR investigating UK restricted grazing methods and their impact on equine welfare. I have previously held several part time customer service roles, and had a temporary position as a research technician on a rat welfare project after finishing my masters. As well as horses, other companion animals I have had and loved include gerbils, degus, and a family dog. As a child I knew I wanted to work with animals in some way, and I always had a particular passion for horses and equine behaviour.

I have grown up riding and caring for horses from a young age as my grandma owned horses since before I was born. I attended and helped at a local riding school, and from the age of 14 loaned a horse with a friend for several years. When I was 15 my grandma suffered an injury which meant her two ponies were moved to the same yard as my loan horse, where my family and I cared for them. In effect they became my own ponies. I cared for them into retirement and until I had to make difficult and painful euthanasia decisions for them both, eight and 10 years later respectively. I felt especially underprepared for both the emotional and practical aspects of the first euthanasia decision I made. My experience in the equine veterinary sector is primarily as a horse owner and client. As well as making end-of-life decisions for my own horses, I have observed others at my yard making these decisions for theirs, and I have also seen first-hand examples of delayed euthanasia and the impact it has on equine welfare. These experiences have given me a valuable understanding of how challenging and distressing end-of-life decisions are for owners. In addition, my undergraduate and postgraduate degrees have given me insight into the struggles veterinary staff also face when making euthanasia decisions with owners. I was initially drawn to my PhD project as I wanted to contribute to work that could help other horse owners prepare for, and be supported through, one of the most difficult decisions they are likely to face as an owner.

Before undertaking the qualitative analysis for my PhD, I attended a three-day Oxford Qualitative Course on conducting and analysing qualitative interviews. My supervisory team is a mix of veterinary surgeons with equine specialisms, and qualitative researchers from the human palliative health care setting. Having a mixed research team has allowed different perspectives to be considered during data analysis. My personal experiences may have influenced my interpretation of the qualitative data I collected, which I aimed to reflect on during analysis. Double coding transcripts and gaining insight and advice from my supervisors has added additional perspectives, to create a more comprehensive analysis.

Relationships were not established with participants in this study prior to data collection. A representative for each establishment was contacted to arrange a suitable time to conduct the focus group. However, one participant was known to the researcher through previous discussions about equine welfare research. Participants were told that the purpose of the research was to investigate their experiences of equine end-of-life decision-making. The primary researcher's role as a PhD student was described, and that the aim was to use findings from this study to develop resources to support end-of-life and euthanasia decision-making.
